# The way of SARS-CoV-2 vaccine development: success and challenges

**DOI:** 10.1038/s41392-021-00796-w

**Published:** 2021-11-09

**Authors:** Yetian Dong, Tong Dai, Bin Wang, Lei Zhang, Ling-hui Zeng, Jun Huang, Haiyan Yan, Long Zhang, Fangfang Zhou

**Affiliations:** 1 School of Medicine, Zhejiang University City College, Hangzhou, 310015 Zhejiang China; 2 Life Sciences Institute and Innovation Center for Cell Signaling Network, Zhejiang University, Hangzhou, Zhejiang 310058, China; 3 Department of Orthopaedic Surgery, The Third Affiliated Hospital of Wenzhou Medical University, Rui‘an, China; 4 Institutes of Biology and Medical Science, Soochow University, Suzhou 215123, China

**Keywords:** Vaccines, Infection

## Abstract

Severe acute respiratory syndrome coronavirus 2 (SARS-CoV-2) is the causative agent of coronavirus disease 2019 (COVID-19). To halt the pandemic, multiple SARS-CoV-2 vaccines have been developed and several have been allowed for emergency use and rollout worldwide. With novel SARS-CoV-2 variants emerging and circulating widely, whether the original vaccines that were designed based on the wild-type SARS-CoV-2 were effective against these variants has been a contentious discussion. Moreover, some studies revealed the long-term changes of immune responses post SARS-CoV-2 infection or vaccination and the factors that might impact the vaccine-induced immunity. Thus, in this review, we have summarized the influence of mutational hotspots on the vaccine efficacy and characteristics of variants of interest and concern. We have also discussed the reasons that might result in discrepancies in the efficacy of different vaccines estimated in different trials. Furthermore, we provided an overview of the duration of immune responses after natural infection or vaccination and shed light on the factors that may affect the immunity induced by the vaccines, such as special disease conditions, sex, and pre-existing immunity, with the aim of aiding in combating COVID-19 and distributing SARS-CoV-2 vaccines under the prevalence of diverse SARS-CoV-2 variants.

## Introduction

A novel coronavirus, severe acute respiratory syndrome coronavirus 2 (SARS-CoV-2), initiated the coronavirus disease 2019 (COVID-19) pandemic in 2019^1–4^. The global spread of COVID-19 results in the devastating loss of lives and economic well-being. Although control measures such as the use of facemasks, social distancing, and isolation play a role in limiting the transmission of SARS-CoV-2, they cannot impede the spread of COVID-19. Thus, vaccines are developed and rollout globally to reduce the morbidity and mortality associated with COVID-19, with several vaccines granted an Emergency Use Authorization in some countries.

SARS-CoV-2 belongs to the *Betacoronavirus* genus and encodes multiple non-structural proteins (nsp; nsp1–nsp10 and nsp12–nsp16), four structural proteins (membrane (M), envelope (E), nucleocapsid (N), and spike (S) proteins), as well as eight accessory proteins^5^. The SARS-CoV-2 S protein is essential for successful invasion of the human body and consists of two subunits; S1, which binds to the angiotensin-converting enzyme II (ACE2), and S2, which is responsible for membrane fusion^6–8^. The S1 subunit is further divided into an N-terminal domain (NTD) and a receptor-binding domain (RBD). Notably, some of the nucleic, vector, and subunit vaccines focus on the viral S protein, whereas inactivated and live-attenuated vaccines are based on the whole virus^9^.

As of 23 September 2021, 121 potential vaccine candidates are in clinical trials and a further 194 candidates are in preclinical testing. Several vaccines, like BNT162b2 and mRNA-1273, exhibited high efficacy in phase 3 clinical trials. However, the emergence of novel circulating mutants has raised concerns about the efficacy of these vaccines. SARS-CoV-2 variants, such as the alpha and beta variants, have spread fast and aggravated the pandemic^10,11^. Thus, a cohort of scientists are exploring whether the SARS-CoV-2 variants impair the neutralization of convalescent serum or current vaccines. Moreover, the immune changes in individuals after natural infection or vaccination are being monitored to better understand the kinetics of immune responses against SARS-CoV-2. In this review, we presented mutational hotspots, the characteristics of SARS-CoV-2 variants, and their abilities to resist neutralization. We also summarized the changes in an individual’s immunity after being infected or vaccinated and discussed the factors that might influence vaccine efficacy. We hope our review will offer clues for exploring the mechanisms used by SARS-CoV-2 variants to evade the vaccine-induced immunity, as well as aid in the distribution of SARS-CoV-2 vaccines, especially to those with a high risk of COVID-19.

### Mutational hotspots of SARS-CoV-2

**Fig. 1:**
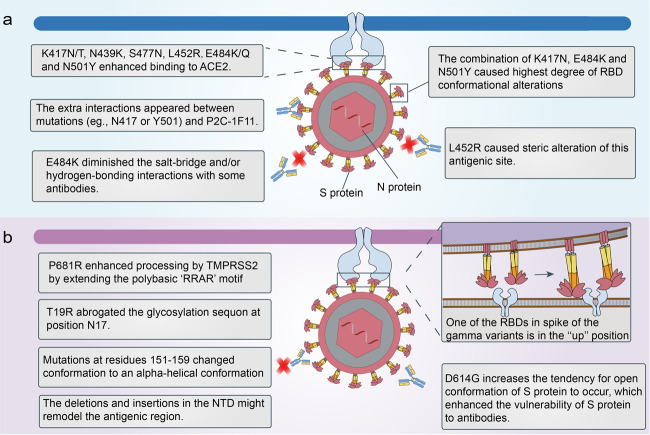
Possible neutralization evasion mechanisms of severe acute respiratory syndrome coronavirus 2 (SARS-CoV-2) variants. **a** The mechanisms of mutations in the receptor-binding domain (RBD) to influence the binding of spike protein to antibodies. **b** The mechanisms of mutations in an N-terminal domain (NTD) to influence the binding of S protein to antibodies. Besides, the gamma variants adopted a conformation with one of RBDs in the “up” position, promoting binding to ACE2 and evasion of some antibodies

The SARS-CoV-2 variants carry a distinctive constellation of mutations and some mutations are of virological importance. The epitopes in RBD account for ~90% of the neutralizing activity of sera from individuals previously infected with SARS-CoV-2^12^. Mutations in the RBD of SARS-CoV-2 variants influence the neutralization activity of antibodies in diverse ways (Fig. 1). The E484K mutation, which occurred in both the beta and gamma variants, diminished the salt-bridge and/or hydrogen-bonding interactions with some antibodies (e.g., BD368-2, P5A-1B9, P2B-2F6, and CV07-270), rendering these antibodies ineffective against these two variants^13^. The E484K mutants also showed resistance to the C121 or C144, 2B04, 1B07, REGN-10989, REGN-10934 antibodies, and polyclonal human convalescent sera^14–17^. Although E484K lowered the neutralizing activity of antibody P2C-1F11, there were additional interactions between N417 or Y501 mutations and P2C-1F11, partly resulting in the retained binding and neutralization of P2C-1F11 against SARS-CoV-2 variants containing the K417N/E484K/N501Y mutations^13^. Some mutations also enhanced binding affinity to human ACE2, which may diminish the binding and neutralizing activities of antibodies. N439K was a prevalent mutation with increased ACE2-binding avidity and reduced some monoclonal antibody and polyclonal serum-mediated neutralization^18^. S477N, E484K, and N501Y, which were present in the alpha, beta, and gamma variants, were also able to enhance binding affinity to ACE2, resulting in the increased transmissibility of those variants in the population^19–21^. E484K or N501Y mutations alone were found to increase the affinity of the RBD to ACE2, whereas the combination of K417N, E484K, and N501Y caused the highest degree of RBD conformational alterations, which may perturb the antigen recognition^22^. The L452R mutation, included in the S protein of the delta and kappa variants, increased viral replication kinetics compared with the wild-type virus^23^. The L452R mutation also impaired neutralization mediated by some clinical antibodies due to steric alteration of this antigenic site that was incompatible with binding^24^.

Apart from mutations in viral RBD, mutations presented in other domains of S protein also have a profound impact on the neutralizing activity of vaccines or neutralizing antibodies (NAbs) (Fig. 1). One mutation in the S protein (D614G) emerged early in the pandemic and spread rapidly through Europe and North America. SARS-CoV-2 variants carrying the D614G mutation have increased infectivity and transmissibility rates^25,26^ and enhanced the vulnerability of the S protein to vaccine-induced neutralization^27,28^, as the D614G mutation increases the tendency for the open conformation of S protein to occur^27^. Therefore, the D614G mutation may not be a barrier to current vaccine development. SARS-CoV-2 utilizes host cell protease transmembrane protease serine 2 (TMPRSS2) for priming of the S protein and mediating virus–host membrane fusion^8^. P681R was at the S1–S2 cleavage site and enhanced processing by TMPRSS2 by extending the polybasic ‘RRAR’ motif, which led to higher viral loads and increased transmission^29,30^. The deletions, insertions, or other mutations such as L18F, D80A, D253G/Y, R246A, S255F, and the Y144 deletion in the NTD potentially aid in the resistance of SARS-CoV-2 variants against antibodies, and the deletions and insertions may remodel the antigenic region^31–33^. The L18F substitution was present in some variants of the beta and gamma variants, which might partly account for these two lineages’ ability to escape neutralization. The T19R substitution, present in the kappa variant, abrogated the glycosylation sequon at position N17, which might influence the neutralization of NAbs^24^. Another mutation in the NTD of the delta variants caused the unexpected remodeling: residues 151–159 adopted an alpha-helical conformation while this segment was β-stranded without this mutation/deletion^24^. The degree to which the SARS-CoV-2 variants were resistant to the antibodies was shown to be correlated with the Y144 deletion and 242–244 deletion in the NTD^13^. Further studies are expected to explore more mechanisms enabling the evasion of novel variants.

### SARS-CoV-2 variants

#### Variants of concern

**Fig. 2:**
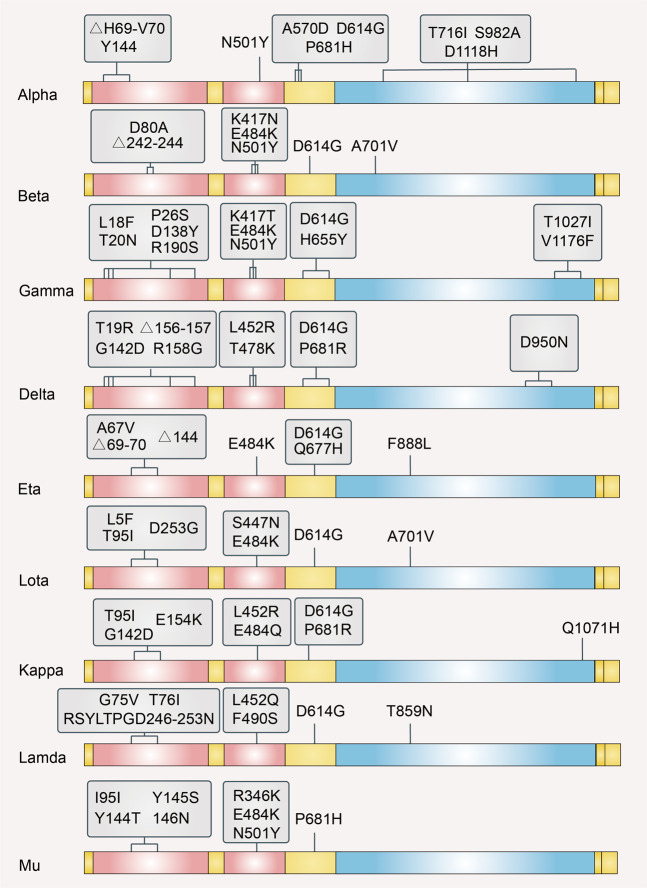
Illustration of common mutations in the S protein sequence of different SARS-CoV-2 variants of concern and interest

Rapidly spreading SARS-CoV-2 variants appeared in the UK, South Africa, China, and other regions. Given the risk on global public health, some variants are classified as Variants of Concern (VOCs) (Fig. 2). VOCs are correlated with increased transmissibility, increased virulence, and a decrease in the effectiveness of public health and social measures or available diagnostics, vaccines, and therapeutics. The World Health Organization (WHO) Weekly Epidemiological update on 2 September 2021, described four VOCs, namely Alpha, Beta, Gamma, and Delta.

The alpha variant was initially detected in the UK. It was associated with higher transmissibility and increased mortality relative to previous circulating forms of virus variants^34–36^. The key mutations that we mentioned above like N501Y helped the virus evade the neutralization of antibodies. The alpha variant also upregulated the expression of ORF6 and ORF9b to halt the host’s innate immunity^37^. It can be neutralized by the majority of serum samples from participants vaccinated with BNT162b2, mRNA-1273, and NVX-CoV2373, and the neutralizing titers were reduced by approximately two-fold^38–42^. ChAdOx1 nCoV-19 was also sufficient to provide protection against the alpha variants^43^. The geometric titers (GMTs) of NAbs against the alpha variant did not change markedly in the BBIBP-CorV vaccine serum samples but reduced more (by a factor of 0.5) in the CoronaVac vaccinee serum samples, compared with the GMTs against the wild-type virus^44^.

The beta variant firstly emerged in the Nelson Mandela Bay area of Eastern Cape Province, South Africa^45^. Compared with the wild-type virus, a notable decrease in neutralization activity against the beta variants was observed for serum samples obtained from individuals receiving the BBIBP-CorV^44^, CoronaVac^44^, mRNA-1273^41,46^, NVX-CoV2373^46^, and BNT162b2 vaccines^41^. In addition, the efficacy of the ChAdOx1 nCoV-19 vaccine against the beta variant was 10.4%, indicating its poor protective capability^47^. Despite a reduction in neutralization activity, the activity induced by BNT162b2, BBIBP-CorV, and ZF2001 could still neutralize the beta variants^48,49^. Besides, NAb titers against the beta variants after vaccination were a bit lower than those against other variants of interest (VOIs)^50,51^. A single dose of Ad26.COV2.S could elicit NAbs and T-cell responses against alpha, beta, gamma, delta, kappa, and epsilon variants, which were durable for eight months and NAb titers against beta variants were a bit lower than those against other variants, suggesting that the beta variant had strong ability to evade the immunity^50^. In addition, CD4^+^ and CD8^+^ T-cell responses in COVID-19 convalescent individuals vaccinated with mRNA vaccines (mRNA-1273 or BNT162b2) are not significantly impacted by mutations found in the SARS-CoV-2 variants, as they are capable of recognizing peptides derived from the SARS-CoV-2 Wuhan ancestral sequence and the alpha, beta, gamma, and delta variants^52^. Although some SARS-CoV-2 variants could partially escape the antibodies generated by natural infection or vaccination, T cells might still be able to target these variants.

The gamma variant was first identified in Brazil, estimated to be 1.7- to 2.4-fold more transmissible than other local SARS-CoV-2 lineages^53^. The gamma variant has 10 spike mutations: L18F, T20N, P26S, D138Y, and R190S in the NTD; K417T, E484K, and N501Y in the RBD; and D614G and H655Y^54^. The spike of the gamma variant adopted a confirmation that one of the RBDs was in the “up” position, facilitating binding to ACE2 (Fig. 1b)^54^. The gamma variant was completely or partly resistant to neutralization by various therapeutic monoclonal antibodies targeting the RBD (e.g., 2-15, REGN 10933, LY-CoV555, CB6, and C121) or the NTD (e.g., 2-17, 4-18, 4-19, and 5-7)^54^. Compared to the wild-type virus, neutralization of the gamma variant significantly declined (6.7-fold for BNT162b2 and 4.5-fold for mRNA-1273)^41^. In addition, serum from individuals vaccinated with CoronaVac had significantly lower neutralization efficiency against the gamma variant, relative to the wild-type virus^55^. The gamma variant and beta variant both have K417T, E484K, and N501Y in the RBD^54,56^. The independent appearance of the same combination of mutations in geographically distinct lineages suggests a process of convergent molecular adaptation. However, the gamma variant was less resistant to naturally acquired or vaccine-induced antibody responses than the beta variant, partly owing to the influence of mutations outside the RBD^54,56^.

The delta variant was first reported in India and now is becoming globally dominant^57^. It is divided into clades A-E, with clade D much prevalent around the world^58^. The clade D is featured by many non-synonymous mutations, which occurred frequently in ORF1a/b, T140I in ORF7b, and G215C in N protein^58^. The delta variant was estimated to be up to 50% more transmissible than the alpha variant^59^. The S protein of the delta variant mediated robust entry into lung cells compared with the S protein of wild-type virus and drove the cell-to-cell fusion, which was associated with P681R in the cleavage site^60^. Compared to the alpha variant, the delta variant caused a higher hospital admission or emergency care attendance risk, indicative of the high virulence^61^. Sera from BNT162b2-vaccinated individuals were three- to five-fold less potent against the delta variants than that against the alpha variants^62^. Moreover, the effectiveness was ~88% with two doses of the BNT162b2 vaccine and approximately 67% with two doses of the ChAdOx1 nCoV-19 vaccine^63^. The effectiveness of two-dose SARS-CoV-2 vaccines (BNT162b2 or ChAdOx1 nCoV-19) was lower to a small degree among persons with the delta variants than among those with the alpha variants^62,63^. Additionally, full doses of inactivated vaccines (CoronaVac or BBIBP-CorV) were still effective against the delta variant infection, with the adjusted real-world effectiveness reaching 59.0%^64^.

### VOI

VOIs are defined as variants with genetic changes that may enhance transmissibility or virulence, evade detection, and affect the effectiveness of therapeutics. Based on the recent update by the WHO, five SARS-CoV-2 VOIs have been identified, namely eta, lota, kappa, lambda, and mu (Fig. 2). The information concerning VOIs has not been characterized as thoroughly as VOCs.

The eta variant born key spike mutations: A67V, Δ69/70, Δ144, E484K, D614G, Q677H, F888L. The lota variant had mutations in S protein: L5F, T95I, D253G, S477N, E484K, D614G, A701V. BNT162b2 vaccine-elicited antibodies efficiently neutralized the eta variant with the GMT of NAbs against the eta variant similar to that against the alpha variants, which might be due to the E484K that increased the affinity of RBD for ACE2^65^. Two doses of mRNA-1273-induced serum samples could neutralize the lota variant, although the activity reduced over time^51^. The lota variant has modest levels of resistance to a convalescent or vaccine sera and the level of resistance sat between that of the alpha variant and the beta variant^51,66^.

The kappa variant originated from India. Unlike the alpha variant, the RBDs of the delta and kappa variants interacted with ACE2 with comparable affinity to the wild-type RBD^24^. Hence, the kappa variant might emerge due to antibody-mediated selective pressure. The S protein of the kappa variant contained several mutations: G142D, E154K, L452R, E484Q, D614G, P681R, and Q1071H. The kappa variant was less susceptible to neutralization by sera from mRNA-1273- or BNT162b2-vaccinated individuals, relative to the wild-type virus and most sera samples from vaccinated individuals were still capable of neutralizing the kappa variant^67^.

The lambda variant is now spreading widely in South America. The S protein of the lambda variant harbored six mutations (i.e., G75V, T76I, L452Q, F490S, D614G, and T859N) and a 7-amino-acid deletion (i.e., RSYLTPGD246-253N)^68^. The S protein of the lambda variant was infectious due to the T76I and L452Q mutations. Moreover, the RSYLTPGD246-253N mutation was shown to be highly correlated with the outbreak of the lambda variant in South America. RSYLTPGD246-253N mutations, L452Q and F490S, accounted for evasion from NAbs and the lambda variant was susceptible to special antibodies that could mediate viral infection^68^.

The mu variant emerged in Colombia in early 2021. It contained several substitutions in S protein, including the amino acid changes I95I, Y144T, Y145S and the insertion 146 N in the NTD, R346K, E484K, and N501Y in the RBD and P681H in the S1/S2 cleavage site of the S protein^69^. Sera from BNT162b2-vaccinated persons all neutralized the mu variant robustly^70^. Up to now, rare evidence has revealed the impact of the novel variant on the efficacy of SARS-CoV-2 vaccines or clinical antibodies, which warrants further study. The ongoing evolution of SARS-CoV-2 necessitates continuous monitoring of vaccine efficacy against the variants and new vaccine preparations may be required to halt the variants circulating globally.

### Progress in SARS-CoV-2 vaccine development

**Fig. 3:**
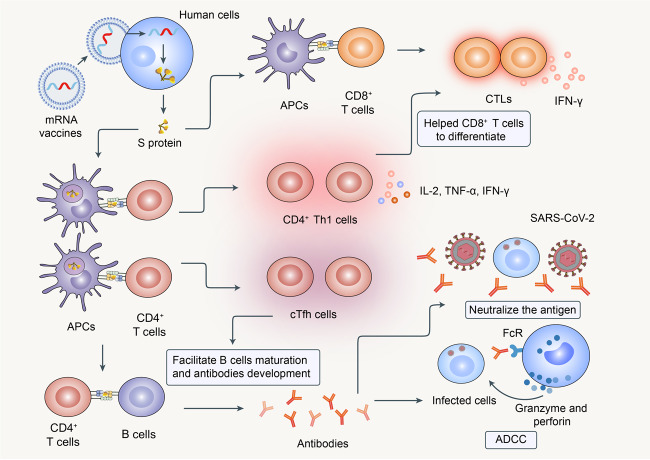
The immune responses induced by mRNA vaccines. The mRNA vaccines encoding the S protein were inserted into human cells and recognized by antigen-presenting cells (APCs). They elicited robust CD8^+^ and Th1-type CD4^+^ T-cell responses. CD4^+^ Th1 cells promoted CD8^+^ T-cell expansion and differentiation through cytokine (e.g., interleukin [IL]−2) production and expressed interferon (IFN)-γ, tumor necrosis factor (TNF)-α, and IL-2. CD8^+^ T cells also expressed IFN-γ. The circulating T follicular helper (cTfh) cells facilitated B-cell maturation and high-affinity antibody production. The vaccines also elicited robust immune responses and promoted antibody-dependent cellular cytotoxicity (ADCC) activity

Hundreds of SARS-CoV-2 vaccine development programs have been initiated since the COVID-19 pandemic broke out, with some vaccines approved for widespread use. The primary and secondary outcomes from phase 3 or 4 clinical trials of several SARS-CoV-2 vaccines are published, which we summarize in Table 1. Inactivated vaccines are traditional forms of vaccines and many developers also adopt this method to construct SARS-CoV-2 vaccines, such as CoronaVac^71,72^, BBIBP-CorV^73^, BBV152^74^, the inactivated vaccines developed by Sinopharm (Wuhan)^75,76^ and the vaccines developed by Chinese Academy of Medical Sciences^77^. A part of nucleic, vectored, protein subunit vaccines is designed based on S protein of SARS-CoV-2. The mRNA vaccines developed by Pfizer/BioNtech (BNT162b2) and Moderna (mRNA-1273) are at the forefront of vaccine development. Both are lipid nanoparticle–encapsulated mRNA-based vaccines that encode the prefusion stabilized full-length S protein of SARS-CoV-2. BNT162b2 elicited dose-dependent neutralizing activities in both young and old adults^78^. The vaccine also increased antibody-dependent cellular cytotoxicity activity in naive and previously infected individuals^79^. Moreover, it elicited robust antigen-specific CD8^+^ and Th1 CD4^+^ T-cell responses against S1 and S2 subunits of S protein; the latter response was dominant^79,80^. CD4^+^ Th1 cells promote CD8^+^ T-cell expansion and differentiation through the production of cytokines, such as interleukin (IL)-2^81,82^. CD4^+^ T cells co-expressing CD40L, interferon (IFN)-γ alone, or in combination with other molecules, such as tumor necrosis factor (TNF)-α, CD107a, and IL-2, and CD8^+^ T cells expressing IFN-γ alone or combined with CD107a were increased in previously infected individuals, compared with naive individuals after vaccination^79^. In addition, the S-specific circulating T follicular helper (cTfh) cells were observed after vaccination with BNT162b2^79^, which could facilitate B-cell maturation and high-affinity antibody production in the germinal center of the secondary lymphoid organ (Fig. 3)^83^. The mRNA-1273 vaccine also showed high efficacy of 94.1% in preventing COVID-19 in phase 3 trial^84^. INO-4800^85^ and ZyCOV-D vaccine^86^ are DNA vaccines that target the full-length S protein, both of which could elicit NAbs and Th1-biased cellular immune responses in vaccinees. Vectored vaccines are another platform of SARS-CoV-2 vaccines. ChAdOx1 nCoV-19^87–91^, Gam-COVID-Vac^92^, Ad26. COV2. S^93^, and Ad5-nCoV^94^ all belong to the vectored vaccines, although they use different vectors to express the S protein. Among protein subunit vaccines, NVX-CoV2373^95–97^, ZF2001^98^, and the SCB-2019 vaccine^99^ entered the phase 3 trial. CoVLP is a virus-like particle vaccine, produced by transient transfection of *Nicotiana benthamiana* plants. AS03-adjuvanted CoVLP elicited NAb titers which were ~10-times higher than those in convalescent serum as well as balanced IFN-γ and IL-4 responses^100^. The mRNA vaccine BNT162b2 is studied more deeply than others and further trials are expected to explore the detailed mechanisms of how different platforms of vaccines provide protection from COVID-19.

**Table 1:** The development of vaccine candidates in phase﻿ 3 or phase 4 clinical stage

Vaccine platforms	Name	Developers	Number of doses and vaccination schedules	Vaccine safety	Vaccine-induced immunity	Vaccine efficacy	Reference
Inactivated vaccines	CoronaVac	Sinovac Research and Development Co.	2 (Day 0 + 14)	Adverse reaction within 28 days after injection occurred in 29-33% of adults aged 18–59 years after the days 0 and 14 schedules and 20% of individuals aged 60 years and older after the days 0 and 28 schedules in the 3 μg group.	The seroconversion rates were 92% in adults aged 18–59 years after the days 0 and 14 schedules and 98% in adults aged 60 years and older after the days 0 and 28 schedules in the 3 μg group.	50% (Brazil) 65% (Indonesia) 67% (Chile) 84% (Turkey)	^71,72^
	BBIBP-CorV	Sinopharm/China National Biotec Group Co/Beijing Institute of Biological Products	2 (Day 0 + 21)	At least one adverse reaction within the first 7 days was reported in 18% of individuals in the 4 μg group after days 0 and 21 schedules.	Robust humoral responses were induced in all vaccine recipients on day 42.	78.1%	^73^
	BBV152	Bharat Biotech	2 (Day 0 + 14)	20% of participants had a solicited local or systemic adverse reaction in the 3 μg Algel-IMDG group.	The seroconversion rates of NAbs on day 56 were 92.9% in the 3 µg Algel-IMDG groups, with more Th1 cytokines observed.	78%	^74^
	Inactivated SARS-CoV-2 vaccine	Institute of Medical Biology/Chinese Academy of Medical Sciences	2 (Day 0 + 28)	23% of participants had adverse reactions within the 28 days.	The seroconversion rates of the NAbs reached at least 80%.	/	^77^
	Inactivated SARS-CoV-2 vaccine	Sinopharm/China National Biotec Group Co/Wuhan Institute of Biological Products	2 (Day 0 + 21)	Adverse reactions in 7 days after each injection occurred in 19.0% of participants who received 5 μg/dose on days 0 and 21.	The GMTs of NAbs were 247 at day 14 after 2 injections in participants receiving a vaccine on days 0 and 21.	72.8%	^75,76^
RNA vaccines	mRNA-1273	Moderna/NIAID	2 (Day 0 + 28)	Adverse events related to the vaccine or placebo occurred in 4.5% of participants in the placebo group and 8.2% in the mRNA-1273 group.	After 14 days following the second vaccination, the seroconversion rates were 100% in all participants and the GMTs of NAbs were 1909 µg/ml in younger adults and 1686 µg/ml in older adults.	94.1%	^84^
	BNT162b2	Pfizer/BioNTech/Fosun Pharma	2 (Day 0 + 21)	More BNT162b2 recipients than placebo recipients reported any adverse event (27% and 12%, respectively).	NAbs and CD4+ Th1-biased responses were noticed in most vaccinees after a single dose of the vaccine, with CD8^+^ T cells identified in fewer individuals.	95% (≥16 years old) 100% (12–15 years old) 91.3% (six months after the second dose)	^78–80^
DNA vaccines	INO-4800	Inovio Pharmaceuticals/ International Vaccine Institute/Advaccine (Suzhou) Biopharmaceutical Co., Ltd	2 (Day 0 + 28)	Local and systemic adverse events were reported in 27.5% of participants receiving at least one dose by 8 weeks post one dose.	Was immunogenic in 100% subjects by eliciting either or both humoral or cellular immune responses with increased Th1 phenotype.	/	^85^
	ZyCOV-D	Zydus Cadila	3 (Day 0 + 28 + 56)	25% of participants reported at least one adverse event and no deaths or serious adverse events were reported in Phase 1 of the study.	Elicited NAb titers and Th1 response responses.	67%	^86^
Vectored vaccines	ChAdOx1 nCoV-19	AstraZeneca/University of Oxford	1–2 (Day 0 + 28)	At least one local symptom was reported after the second vaccination with standard-dose (SD) by 76% of participants in the 18–55 years group, 72% in the 56–69 years group, and 55% in the 70 years and older group. At least one systemic symptom was reported after the second vaccination by 65% in the 18–55 years group, 72% in the 56–69 years group, and 43% in the 70 years and older group	Induced antibody responses predominantly of IgG1 and IgG3 subclasses and a Th1-biased response.	62.1% (SD/SD) 90% (lower dose/SD)	^87–91^
	Gam-COVID-Vac (rAd26-S + rAd5-S)	Gamaleya Research Institute/Health Ministry of the Russian Federation	2 (Day 0 + 21)	94.0% of reported adverse events were grade 1 and 0.3% of participants in the vaccine group had serious adverse events.	Cellular immunity was detected in all participants on day 28 and NAbs were detected in 100% of participants on day 42.	91.6%	^92^
	Ad26.COV2.S	Janssen Pharmaceutical	1–2 (Day 0 + 56)	During the 7-day period after the administration of the vaccine, more solicited adverse events were reported by vaccinees aged 18–59 years than by those 60 years and older.	NAbs and CD4+ Th1-biased responses were noticed in most vaccinees several days after a single dose, with CD8^+^ T cells identified in fewer individuals.	66.9%	^93^
	Ad5-nCoV	CanSino Biological Inc./Beijing Institute of Biotechnology	1	Solicited adverse reactions were reported by 74% of participants and severe adverse reactions were reported by 1% of participants in 5 × 10^10^ viral particles dose.	Elicited high humoral and cellular immune responses in most receipts 28 days post-immunization.	74.8%	^94^
Protein subunit vaccines	NVX-CoV2373	Novavax	2 (Day 0 + 21)	After the second vaccination, local reactogenicity was absent or mild in 65% of participants and systemic reactogenicity was absent or mild in 73% of participants in the 5-μg plus Matrix-M1 groups.	Anti-spike IgG and neutralization responses exceeded values in COVID-19 convalescent serum from mostly symptomatic COVID-19 patients.	89.3% (UK) 60.1% (in HIV-negative subjects, South Africa)	^95–97^
	ZF2001	Anhui Zhifei Longcom Biopharmaceutical/Institute of Microbiology, Chinese Academy of Sciences	2–3 (Day 0 + 28 or Day 0 + 28 + 56)	Adverse events reported within 30 days after vaccination were mild or moderate (grade 1 or 2) in most cases (29% of participants in the two-dose 25 μg group; 48% in the three-dose 25 μg group).	Three immunizations with different doses (25 or 50 μg) achieved 93–100% seroconversion of NAbs at day 0, 30, and 60 and induced Th1 and Th2 cytokines.	81.76%	^98^
	SCB-2019	Clover Biopharmaceuticals Inc./GSK/Dynavax	2 (Day 0 + 21)	The incidence of local adverse events was 35% in young adults aged 18–54 years and 34% in old adults aged 55–75 years and the incidence of systemic adverse events was 30% in old adults and 34% in young adults after the second dose.	Elicited high titers and seroconversion rates of binding and NAbs and Th1-biased CD4+ T-cell responses in both younger and older adults.	/	^99^
Virus-like particles vaccines	CoVLP	Medicago Inc.	2 (Day 0 + 21)	74.3% of participants reported more than one solicited adverse event after the first dose.	Elicited NAb titers and balanced IFN-γ and IL-4 responses.	/	^100^

The divergence in efficacy of diverse platforms of SARS-CoV-2 vaccines may be attributed to several factors, such as vaccine design, the SARS-CoV-2 variants, and vaccination timing. Many viral surface proteins exist as trimers in a post-fusion state, which fosters antibody responses that have limited protective ability^101^. To optimize these vaccines, the proline residues are often introduced at key positions of S protein to maintain the trimer in the prefusion form. Previous evidence revealed that the proline mutations were clearly beneficial to the immunogenicity of the MERS-CoV S protein^102^. Besides, introducing furin cleavage-site substitutions into vaccine antigen further increased the yield of S trimers and improved protective capacity. The introduction of two proline substitutions and furin cleavage-site knockout in the vaccine antigen provided strong protection in the animal model^103^. BNT162b2, mRNA-1273, NVX-CoV2373, and Ad26.COV2.S all applied two proline mutations (i.e., K986P and V987P), combined with cleavage-site mutations to the S protein. The efficacy and real-world effectiveness of these vaccines are high, which indicates that these vaccine designs aid in the protection of individuals against COVID-19.

Moreover, as the SARS-CoV-2 variants vary in different regions and times, the efficacy and the real-world effectiveness of the same vaccine tested in different regions and times may vary to some extent. According to the WHO, in phase 3 clinical trials, the inactivated vaccine CoronaVac had an efficacy rate of 50%, 65%, 67%, and 84% in Brazil, Indonesia, Chile, and Turkey, respectively. The efficacy was a bit low in Brazil partly owing to the prevalence of the gamma variants, which robustly hindered the neutralization of NAbs, but the estimated efficacy still exceeded the WHO minimal threshold of 50%^44^. Moreover, the effectiveness of BNT162b2 vaccines against the delta variants 14 or more days after the second dose was only 53.5%, but 75.0% against the beta variants^104,105^. The effectiveness against the beta variants was estimated at a time when most persons in Qatar newly received BNT162b2 vaccines, but effectiveness against the delta variants was estimated at a time when the second dose was given among persons several months earlier^104^. Thus, the low effectiveness against the delta variants among fully vaccinated persons may reflect some waning of BNT162b2 protection over time.

As the titers of NAbs waned over time after vaccination and SARS-CoV-2 variants perturbed the neutralization activity of some vaccines, inoculation of the third dose of vaccines that were already put into emergent use or the novel vaccine especially against the novel SARS-CoV-2 variants were tested whether to increase the neutralization of SARS-CoV-2 variants. Vaccination of the third dose of mRNA-1273 or BNT162b2 robustly triggered the high titers of NAbs against the SARS-CoV-2 variants, including the beta and delta variants^106^. Moreover, inoculation of mRNA-1273.211, a vaccine especially against the beta variant, neutralized the variant more strongly compared with the mRNA-1273^106^. However, current vaccine supplies could save more lives if used in previously unvaccinated populations than if used as boosters in vaccinated populations. WHO also has called for a moratorium on boosting until the benefits of primary vaccination (one-dose or two-dose series of each vaccine) have been made available to more people around the world. Hence, it is still in urgent need to promote that more unvaccinated persons receive the SARS-CoV-2 vaccines.

### Factors that may affect vaccine-induced immunity

#### Special populations

**Fig. 4:**
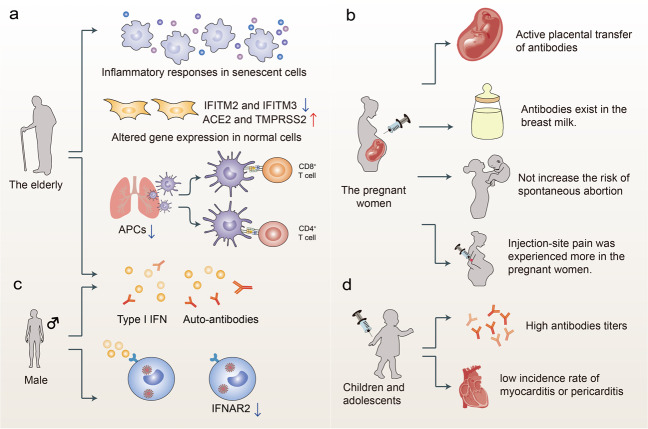
Factors that may affect the immunity induced by the vaccines. **a** The immune responses in the elderly. Inflammatory responses were robust in senescent cells and gene expression changed in normal cells. Immunosenescence reduces the levels of APCs and induces anti-type I interferon (IFN) autoantibodies in the elderly. **b** Vaccination for pregnant women. Vaccination in pregnant women is safe, with injection-site pain being frequently observed and without increasing the risk of spontaneous abortion. Moreover, vaccine-induced antibodies existed in the cord blood of infants and in the mothers’ breast milk. **c** The immune responses in males. Males are prone to elicit anti-type I IFN autoantibodies, and the expression of IFNAR2 reduces. **d** Vaccination for children and adolescents. Vaccination for children and adolescents induced high titers of antibodies, although myocarditis and pericarditis were noticed in some young individuals

Children and adolescents are a critical part of populations. They have a low incidence of SARS-CoV-2 infections and if infected, are asymptomatic or have mild symptoms^107–109^. However, they still play a key role in the SARS-CoV-2 transmission and whether vaccinating them to reach herd immunity has aroused much discussion. Inoculation of inactivated vaccines CoronaVac or BBIBP-CorV on children and adolescents (3–17 years old) was safe, with most adverse events mild or moderate; they also induced high NAbs titers^110,111^. The GMT of NAbs induced by an mRNA vaccine BNT162b2 in children who were 5–11 years of age or 12–15 years of age was non-inferior to that in adolescents aged 16–25 years old, with adverse events similar to those noticed in persons aged 16–25 years old^112^. Further trials are conducted to explore the safety, tolerability, and immunogenicity of BNT162b2 in children 6 months to 11 years of age. The information about the safety, immunogenicity, and efficacy of mRNA-1273 on children and adolescents aged 12–17 years was reported in press releases. The phase 2/3 study showed the vaccine efficacy of 100% starting 14 days after the second dose, with no COVID-19 observed in participants. However, an association between both mRNA vaccines and myocarditis/pericarditis was noticed in younger individuals aged 12–39 years, which requires further investigation (Fig. 4)^113^.

The elderly have higher frequencies of SARS-CoV-2 infections and mortality rates compared with those among young people^107,108,114,115^. A single dose of the vaccine was shown to be insufficient to induce the potent neutralization activity against VOCs in the elderly, whereas two doses of vaccines could neutralize VOCs^116^. The elderly participants also exhibited a clear reduction in somatic hypermutation of class-switched cells and showed low cellular responses^116^. Protection against re-infection was just 47% for those aged 65 years or older ~6 months after the first viral infection, suggestive of immune senescence in the elderly^117^. Senescent cells were hyper-inflammatory in response to pathogen-associated molecular patterns, and antiviral responses decreased whilst expression of viral entry genes, *ACE2* and *TMPRSS2*, increased in non-senescent cells^118^. Like SARS-CoV, SARS-CoV-2 may be capable of evading the innate immune responses, thereby reducing IFN production and limiting T-cell priming^119^. Moreover, professional antigen-presenting cells may be reduced in aged lungs, which also suppresses the prime action of adaptive immune reactions^120^. A recent study showed that patients with autoantibodies against type I IFN that were associated with poor outcomes were slightly older than the rest of the cohort, indicating that anti-type I IFN autoantibodies might increase with age (Fig. 4)^121^. It is also less likely for the elderly to make a coordinated CD4^+^/CD8^+^ T-cell and antibody response against SARS-CoV-2; besides, the lack of naive CD8^+^ and CD4^+^ T cells was linked with aging and COVID-19 disease severity^122^.

WHO has suggested vaccination with SARS-CoV-2 vaccines for pregnant women, with the aim of protecting the newborn during early life through the active placental transfer of antibodies, protecting the mother and the fetus from severe COVID-19 if the infection happens during pregnancy (Fig. 4). Binding and neutralizing antibodies were found in the cord blood of infants that were born to mothers vaccinated with mRNA vaccines and in the mothers’ breast milk, which might provide protection to the infants^123^. Injection-site pain was experienced more frequently while headache, myalgia, chills, and fever were experienced less frequently among pregnant persons than in non-pregnant women^124^. However, no apparent safety concerns were noticed in pregnant women^125^. Besides, vaccination of mRNA vaccines before conception or during pregnancy will not increase the risk of spontaneous abortion^125^. An observational cohort study of 10,861 pregnant women showed 97% effectiveness of BNT162b2 against symptomatic infection in days 7–56 after the second dose, which was similar to the effects estimated in the general population (94% against symptomatic infection), and no severe illness was observed in the vaccinated groups^124,126^. It warrants further investigation to clearly characterize the dynamics of vaccine effectiveness throughout pregnancy and the correlation between vaccination timing and infant protection after birth.

Trials are also conducted to explore the safety and immunogenicity of SARS-CoV-2 vaccines on immune-comprised patients who are more susceptible to viral infection. The immune responses induced by vaccines are diverse in different populations based on their specific diseases, the severity of diseases, and other characteristics. Compared with that in healthy people, seropositivity was significantly lower among immunocompromised patients with solid organ transplant (SOT), autoimmune issues, hematological malignancies, and solid tumors, and the titers of antibodies were much lower in individuals with SOT^127^. The safety and immunogenicity of BNT162b2 were explored in patients with solid- and hematological cancers. Notably, single-dose BNT162b2 had low efficacy in patients with cancer, but the second dose significantly increased immunogenicity in patients with cancer within 2 weeks of a vaccine boost, indicative of the importance of administering the second dose early in patients with cancer^128^. A protein subunit vaccine NVX-CoV2373 was inoculated in medically stable persons infected with HIV, but the total population was too small to determine the efficacy of vaccines for HIV-infected patients. Larger trials are required to test the efficacy of each vaccine on subsets of immune-comprised patients with specific conditions.

#### Sex

Sex affects COVID-19 severity and mortality rates, with higher rates of hospital admissions and deaths in males^129^. The estrogen and testosterone sex hormones regulate immune responses in diverse ways which may affect disease severity^130^. Moreover, social factors might partly account for the difference between females and males, as females might have a higher adherence rate towards safety measures and a higher proportion of females may stay indoors than males. Additionally, ~10% of severe COVID-19 cases in a study had type I IFN autoantibodies, and 94% of those cases were males^121^. A genome-wide association study also reported reduced production of the type I IFN receptor gene, *IFNAR2*, which might lead to severe COVID-19 (Fig. 4)^131^. Moreover, males exhibited higher SARS-CoV-2 neutralization than females, while females had more stable antibody levels than males^132^.

#### Pre-existing immunity

**Fig. 5:**
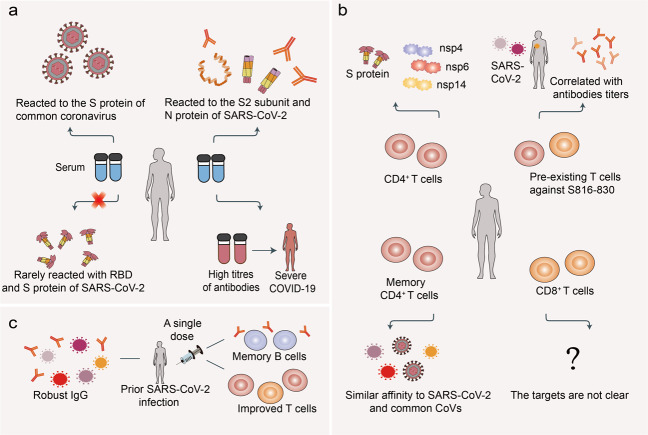
**a** The target of pre-existing antibodies in SARS-CoV-2 unexposed individuals. The serum from SARS-CoV-2 unexposed individuals reacted to the S proteins of common coronavirus, with some antibodies against S2 subunit and N protein of SARS-CoV-2 and rarely against RBD. Moreover, high titers of pre-existing antibodies might correlate with severe COVID-19. **b** The target of pre-existing T cells. The cross-reactive CD4^+^ T cells mainly recognized the S protein and some non-structural proteins, but target proteins of CD8^+^ T cells are unknown. Pre-existing T cells against S816-830 correlated with levels of anti-SARS-CoV-2 antibodies. Furthermore, memory CD4^+^ T cells had a similar affinity to SARS-CoV-2 and common cold coronavirus. **c** Vaccination for individuals with prior-SARS-CoV-2 infection. A single dose of SARS-CoV-2 vaccine induced robust humoral and cellular immune responses in previously SARS-CoV-2-infected individuals

Four pre-emergent human coronaviruses are known causative agents of common cold, including the alphacoronaviruses, HCoV-NL63 and HCoV-229E, and betacoronaviruses, HCoV-OC43 and HCoV-HKU1. The early development of SARS-CoV-2-specific immunity correlated with pre-existing common coronavirus humoral immunity in individuals (Fig. 5)^133^. Most serum samples from SARS-CoV-2 negative control subjects reacted well to the S protein of common coronavirus, but rarely cross-reacted with the SARS-CoV-2 RBD and S protein, with some antibodies cross-reacting with conserved antigens such as the S2 subunit and N protein^134,135^. It is plausible that the S protein, RBD, in particular, is the main target of antibodies, and SARS-CoV-2 shows low homology to the four endemic human coronaviruses RBD region^136^. However, higher titers of IgG against the HCoV-OC43 S protein were observed in patients with relatively severe COVID-19, suggesting that pre-existing immunity has the possibility to accelerate COVID-19 disease progression^137^. CD4^+^ T-cell immune responses have a major role in the cross-reactive immune memory against SARS-CoV-2 (Fig. 5); they can modulate disease severity, reduce viral load, and limit the duration of the disease. Several studies detected CD4^+^ T cells which prominently recognized the S protein and recognized other non-structural proteins, such as nsp14, nsp4, and nsp6 in ~20–60% healthy individuals not exposed to SARS-CoV-2^138–141^. In addition, cross-reactive CD4^+^ T cells with a predominantly Th1 memory phenotype from unexposed individuals mainly recognized the C-terminal epitopes in the S protein^139^. The frequency of pre-existing T cells especially against S816-830 correlated well with titers of anti-SARS-CoV-2 IgG antibodies especially against S1 subunit, highlighting the importance of the spike-cross-reactive T cells in COVID-19^142^. Moreover, the pre-existing memory CD4^+^ T cells cross-reacted with comparable affinity to SARS-CoV-2 and the common cold coronaviruses^139,140^. CD8^+^ T-cell immune responses were observed in a few unexposed donors, but the targeted SARS-CoV-2 proteins were unclear, indicating that CD8^+^ T-cell cross-reactivity is not widespread^138^. The effect of pre-existing SARS-CoV-2 cross-reactive T cells on disease progression, herd immunity threshold, and performance of COVID-19 candidate vaccines remains to be determined in larger cohorts.

In addition, a single dose of BNT162b2 or mRNA-1273 in previously infected individuals induced IgG antibody responses against S protein that was similar to or higher than those noticed after two vaccine doses in recipients without a previous natural SARS-CoV-2 infection (Fig. 5)^143–147^. Moreover, the second vaccine dose did not cause the increase in neutralizing titers in the previously infected subjects^144,146^. After a single BNT162b2 dose, the level of anti-S-protein IgG was higher among older adults previously infected with COVID-19 than in those without a prior COVID-19 infection, indicating that one dose of the vaccine might be adequate in previously infected elderly individuals^148^. After one dose of BNT162b2, individuals with a prior infection demonstrated improved T-cell responses, antibody-secreting memory B-cell response to S protein, and NAbs against the alpha and beta variant^149^. Interestingly, human leukocyte antigen polymorphisms influenced the extent of T-cell immunity elicited by the alpha and beta variant spike mutations^149^.

### The maintenance of immune responses post infection or vaccination

The duration of immune responses post infection and vaccination plays a pivotal role in protection against COVID-19 or re-infection. Hence, it is necessary to surveil the antibodies and the major lymphocytes, namely B cells, CD4^+^ T cells, and CD8^+^ T cells, as declining adaptive immune responses may put the recovered individuals at risk of re-infection. Anti-SARS-CoV-2 IgA and IgM titers decline faster early on, partly accounting for the decline of overall antibody titers^150,151^. Anti-SARS-CoV-2 IgG did not decline drastically in both serum and saliva for at least three months post-symptom onset (PSO), but a pronounced drop in serum NAbs was identified 105–115 days PSO^150^. However, another study reported that titers of IgM and IgG against the RBD of SARS-CoV-2 decreased significantly over 6 months, with IgA being less affected^152^. Other follow-up studies also noticed the declines of IgG and NAb responses^153–155^. Moreover, antibodies against SARS-CoV-2 were maintained for at least 4 months^156,157^. A longitudinal cross-sectional study demonstrated that NAb levels did not decline markedly for ~9 months in some of the participants who were positive for pan-immunoglobulins at baseline, with NAb titers lower in asymptomatic individuals than in symptomatic individuals^158^. The different sampling intervals and subjects with varying disease severity may partly account for the discrepancy of the duration of the antibody response. The duration of immune responses in individuals post-vaccination was also monitored. Based on follow-up data from the phase 1 trial of the BBV152 vaccine, a marginal decline in NAb titers was observed at day 104 (3 months after the second dose)^74^. For mRNA-1273, binding antibody and NAb activities remained high in groups with young and old individuals six months after the second dose (100 μg) of vaccines^159^. Although there is a gradual decrease of NAb titers resulting in reduced protection against a SARS-CoV-2 infection, these titers are still enough to combat severe infection if not lower than the minimum because the neutralization levels were estimated to be 20.2% and 3% of the mean convalescent level for 50% protection against a detectable SARS-CoV-2 infection and severe infection, respectively^160^.

Notably, the S protein and RBD-specific memory B cells were detected in the majority of COVID-19 cases and memory B-cell frequency steadily increased during the first 4–5 months PSO^151,152,161,162^. The neutralizing activity of RBD-targeted antibodies and the number of RBD-specific memory B cells remained stable from 6–12 months PSO, and vaccination increased the humoral responses^163^. However, anti-N protein antibody titers decreased markedly during this period^163^. Memory B cells exhibited clonal turnover after 6.2 months and produced antibodies with increased somatic hypermutation, potency, and resistance to RBD mutations^152^. Maturation over months increased the affinity, potency, and breadth of antibodies and restricted viral escape. These properties result from the substitutions of antibodies that generated new interactions with the RBD^164^. It indicates that increasing antibody variability through repeated antigen exposure may inhibit the escape of VOCs. Therefore, it is significant to give a booster dose of vaccine. Charting of memory B-cell receptor-encoded antibodies from COVID-19 convalescent subjects identifies seven major epitopic regions of S protein. SARS-CoV-2 variants could escape some of the potent neutralizing antibodies, but many retain affinity^165^. Furthermore, long-living bone marrow plasma cells (BMPCs) were detected in some convalescent patients 7–8 months after infection, and S protein-specific IgG BMPCs exhibited a moderate correlation with IgG titers^162^. This might account for the slow decay rate in antibody titers after a period post infection, as the sources of antibody production transited from short-lived plasmablasts to BMPCs^166^.

T-cell immune responses are significant for protection against viral infections and several studies reported changes in cellular immune reactions PSO. One study found T-cell responses to at least one SARS-CoV-2 protein in 95% of participants 6 months post infection, which was 50% higher in individuals with an initial symptomatic infection than those with an asymptomatic infection^167^. This indicates that the magnitude of this response may be correlated with the severity of the primary infection. Of note, memory CD4^+^ and CD8^+^ T-cell frequencies were lower in hospitalized cases compared with non-hospitalized cases^151^. Besides, CD4^+^ T-cell responses outnumbered the CD8^+^ responses from 6 months post infection onwards^151,167^. However, a follow-up study on six discharged participants revealed that a single participant still had a high number of IFN-γ-secreting T cells in response to the N protein, main protease, and the RBD^168^. Another study also discovered that SARS-CoV-2-specific CD4^+^ and CD8^+^ T cells declined with a half-life of 3–5 months^151^. Notably, antibody levels wane faster than T cells. Memory SARS-CoV-T cells that were reactive to the N protein of both SARS-CoV and SARS-CoV-2 were detected 17 years after the initial outbreak of SARS^169^. Furthermore, a study found that SARS-CoV-specific antibodies became undetectable in some individuals within 2–3 years, whereas in others, NAbs were still discovered after 17 years, suggestive of the heterogeneity of immune responses^170,171^. Hence, whether memory T cells for SARS-CoV-2 might have a slower decay than antibodies warrants further study.

Moreover, NAbs were positively correlated to IgG antibody titers and some pro-inflammatory cytokines, including the stem cell factor and monocyte colony-stimulating factor^154^. However, there are contradictory studies indicating the positive and negative correlation of NAbs with the numbers of virus-specific T cells^168,172^. Furthermore, high T-cell responses against N or M proteins at six months correlated with a slow decline in N-specific antibody levels, indicating that these antibody responses may be highly T-cell-dependent, while T-cell responses against S protein were not related to the rate of decline of antibodies against that protein^167^. Although an immune memory of a SARS-CoV-2 infection develops in almost all subjects, the relationships between the individual immune memory compartments are complex. Therefore, the factors that influence the relationship between NAbs and T cells require further clarification.

### Conclusions and perspectives

The COVID-19 pandemic is devastating and is still severe in some parts of the world. Novel SARS-CoV-2 variants have emerged one after another worldwide, some of which have increased the ability to evade immunity conferred by original SARS-CoV-2 vaccines or convalescent serum. Although some vaccines could still neutralize the variants, their efficacy was influenced to a certain degree. Hence, various strategies are suggested to combat the novel variants, such as vaccination of booster dose, developing vaccines specially against VOCs, and developing multi-valent vaccines. Moreover, escape-resistant antibody cocktails have been suggested, including cocktails of antibodies that compete for binding to the same RBD surface but to different escape mutations. Besides, several studies report on how the novel SARS-CoV-2 variants influenced humoral responses but rarely investigated how these variants affected the cellular immune responses. Given that cellular immunity may be critical for halting the SARS-CoV-2 infection, T-cell response to the new variants should be monitored. The waning of antibodies or T cells in individuals after infection or vaccination is another challenge in the way of containing the pandemic. Furthermore, a number of factors like special disease conditions, age, sex, and pre-existing immunity also impact the human immune responses, which offers suggestions that which groups of the population should be prioritized in vaccination. There is a long way to go to achieve herd immunity owing to several obstacles. In conclusion, further research is required to test the efficacy and safety of vaccines under development, optimize them for multiple viral lineages, and increase efforts to promote herd protection.
